# SLM-based PAPR reduction for improved performance of DCO-OFDM LiFi using blind estimation for healthcare monitoring system

**DOI:** 10.1038/s41598-026-43583-9

**Published:** 2026-03-30

**Authors:** Asmaa A. Sharaf, Hussein Seleem, Amany Sarhan, Amira S. Ashour

**Affiliations:** 1 Electronics and Electrical Communications Engineering, Faculty of Engineering, Tanta University, Tanta, Egypt; 2 Electrical Engineering, Faculty of Engineering, Pharos University in Alexandria, Canal El Mahmoudia street, Beside Green Plaza Complex 21648, Alexandria, Egypt; 3 Computer and Control Engineering Department, Faculty of Engineering, Tanta University, Tanta, Egypt; 4 Faculty of Engineering, Elsewedy university of technology, Ramadan City, Egypt

**Keywords:** PAPR reduction, DCO-OFDM, LiFi, Blind channel estimation, Healthcare monitoring, Diseases, Engineering, Health care, Medical research

## Abstract

The necessity for reliable healthcare monitoring following the COVID-19 pandemic has highlighted the limitations of RF-based devices in medical settings. Visible light communication (VLC), which provides inherent security and is resistant to RF interference, is a good alternative. This work proposes a VLC system using DC-biased optical (DCO) orthogonal frequency division multiplexing (OFDM) for resilient, high-speed biomedical data transmission through indoor optical fading channel. In this system, data is modulated using quadrature amplitude modulation (QAM) with order 4 and 16. Four equalization methods; block-type, comb-type, superimposed training (ST), and blind channel estimation (CE); are implemented across three patient positioning scenarios. We integrate blind channel estimation and SLM-based PAPR reduction for a dynamic healthcare LiFi scenario. A comprehensive comparative analysis of CE techniques is conducted under realistic patient positioning (LOS/NLOS) conditions. An analysis of the trade-off between spectral efficiency and energy-to-noise ratio is examined in this context. Simulation results reveal a high Peak-to-Average Power Ratio (PAPR), reaching 15 dB with block-type CE. To mitigate this, Selected Mapping (SLM) is applied with three complex phase sequences, and three real sequences, achieving up to 4 dB PAPR reduction with no Bit Error Rate (BER) degradation. At 28 dB SNR, BER values were $10^{-2}$, $3\times 10^{-2}$, $4\times 10^{-2}$, and $7\times 10^{-2}$ for blind, block-type, comb-type, and ST CE, respectively. Spectral efficiency declines with increased multipath, yet blind CE maintains the highest performance, reaching 0.9 bits/s/Hz with 20 multipath components. Additionally, complex phase vectors in SLM provide an extra 1 dB PAPR gain over real-valued versions.

## Introduction

Recently, Visible light communication (VLC) has been used for several applications in different fields, including healthcare indoor communication, subways identification system, and wavelength division multiplexing (WDM) integrity for developing communication technologies^1,2^. VLC offers numerous advantages compared to RF-based systems, which suffers from limited, non-free RF spectrum, congestion problems, sensitivity to electromagnetic interference, and limited data rates^3^. VLC systems can achieve high data rates in optical communication by utilizing the free and unlicensed optical spectrum, which can serve an unlimited number of users. VLC offers additional advantages, including enhanced security being less susceptible to hacking reduced inter-symbol interference (ISI), and cost-effectiveness. The system uses simple optoelectronic components as light-emitting diode (LED) or Light Amplification by Stimulated Emission of Radiation (LASER) at the transmitter to send data in optical form, and a photodetector (PD) at the receiver for optical to electrical signal conversion^4^. In VLC, at the transmitter, LED intensity is modulated and demodulated at the receiver using intensity modulation direct detection (IM/DD)^5^. Hermitian symmetry must be used at transmitter for transmitting only real values of symbols^6^. LED modulation bandwidth is restricted; hence, VLC commonly uses orthogonal frequency division multiplexing (OFDM), that is one of the common multicarrier modulations (MCM) algorithms and has the ability of reducing ISI^5^. The clipping distortion caused by the nonlinear characteristics of LEDs while preserving the BER is reduced by extending the LED’s linear operating region or reducing PAPR. This will also enhance power and spectral efficiency^7^. PAPR reduction algorithms include Discrete Hartley Transform (DHT), selective mapping (SLM), discrete cosine transforms (DCT), Gaussian matrix (GM), partial transmit sequence (PTS), active constellation extension (ACE), tone injection (TI), Walsh-Hadamard Transform (WHT), and Vander-monde like matrix (VLM)^8^.

On contrast, severe challenges appeared due to transmitting data over a great number of subcarriers. They are constructively combined, peak to average power ratio (PAPR) still high leading to data clipping due to the nonlinear voltage/current characteristic response of the LED at higher power levels^8^. In addition, poor performance is considered a critical challenge, especially with severe fading VLC channels and using traditional equalizers.

Recently, PAPR reduction algorithms for OFDM VLC systems have been researched. Miriyala et al.^7^ designed DCO-OFDM VLC system, using top samples detection and appending (TSDA) for PAPR reduction, the technique is based on using top samples to enhance the PAPR reduction. Several studies^9–11^ proposed OFDM, and Asymmetrically clipped DC-biased OFDM (ADO-OFDM) VLC system, using $\mu$-law logarithmic companding technique at the transmitter frond end, and decompander before the demodulator at the receiver for OFDM signal reconstruction. The results achieved PAPR reduction of 70$\%$, and BER increase by only 0.03. Taha et al.^8^ made a hybrid precoding algorithm, combining VLM with GM technique for the OFDM VLC system to decrease the PAPR, and preserving the BER at the same time. The hybrid system could achieve better performance as compared to DCT&GM, VLM, GM, and DCT, where PAPR has reached 9.5dB. Farid et al.^5^ proposed an OFDM VLC system, using non-distorting precoding techniques, such as DHT, DST, DCT, and VLM for PAPR reduction as well as system performance improvement without affecting the data rate, and BER. From these precoding techniques, there is no requirement for sending side information. The results demonstrated that the PAPR is reduced by 2.17dB, 2.12dB, 1.46dB, and 1.35dB for VLM, DST, DCT, and DHT, respectively. Aydin et al.^6^ proposed DCO OFDM VLC system, in which a novel technique, called multi-point constellation method (MPC) is used for PAPR reduction. The technique is based on two basic steps, at first adding more constellation points, alternative for the existing points, then the second step, in which discrete particle swarm optimization (DPSO) technique is used to choose the constellation points, having the minimum PAPR. The results explained that the increase of the DPSO particles, and their iterations achieve PAPR reduction improvement. Moreover, Zenhom et al.^12^ implemented VLM precoding algorithm with modified O-OFMD techniques like layered asymmetrically clipped optical OFDM (LACO-OFDM), asymmetrically and symmetrically clipping optical OFDM (ASCO-OFDM), FLIP-OFDM, ACO-OFDM, ADO-OFDM, and DCO-OFDM to lower the PAPR factor with enhancing the performance of the system. The method does not require transmitting side information, hence preserving the system’s data rate. The same authors^13^, simulated an ACO OFDM VLC system by applying WHT or DST precoding techniques at the transmitter and frequency domain noise cancelation (FDNC) or time-domain noise cancelation (TDNC) at the receiver. The BER performance remains unchanged for the two receivers. The results indicate performance enhancement by 1.65dB, and 2.97dB for modulation 4QAM, and 1024QAM, respectively, PAPR enhancement by 6.76$\%$, and 20.83$\%$ for WHT, and DST, combined with noise cancellation, respectively, and signal to noise ratio (SNR) enhancement by 14.64$\%$, and 10.10$\%$ for WHT, and DST, respectively. Moreover, some studies used machine learning (ML) and deep learning (DL) for better performance. SALMAN et al.^14^ used ML regressor to optimize DC bias in an DCO-OFDM-VLC system for performance enhancement. They simulated the system at different number of subcarriers and QAM order. The results explained that, at adaptive dc bias, QAM order 4 and SNR 30 dB, BER reached $10^{-5}$, $2\times 10^{-5}$, $4\times 10^{-5}$, and $6\times 10^{-5}$ using 64, 128, 265, and 512 subcarriers, respectively. Li^15^ proposed hybrid technique between convolutional neural network (CNN) and deep neural network (DNN) in VLC system. The study achieved BER enhancement by nearly 4–15 dB through LED nonlinearity mitigation. The previously mentioned studies are summarized in Table 1.

Most previous PAPR reduction methods suffered from complexity, inappropriateness for severe fading VLC optical channels, high PAPR problem, reduced power efficiency and nonlinear distortions in optical components. These are fundamental in continuous, real-time data transmission as biomedical monitoring. In general, they almost supposed LOS VLC channel without fading for simplicity, but that is far away reality, not considering patient position or any other surrounding obstacles for healthcare monitoring application. Furthermore, they used conventional equalizers, which are ineffective for any fading VLC channel situations. Nowadays, most healthcare communication systems use traditional RF technology not able to adopt to VLC. There is a limited research for healthcare applications which motivated the work in this paper. This research gap is mostly due to unresolved technological challenges such as PAPR and channel fading.

**Table 1:** Summary of VLC-OFDM PAPR reduction techniques.

Reference	Technique	Key parameter	Challenges addressed	Contributions	Performance metric
^7^ 2020	TSDA (Top Samples Detection & Appending)	PAPR reduction	High PAPR in DCO-OFDM	Introduced TSDA for improved PAPR reduction	PAPR reduction efficiency
^9^ 2020-21	$\mu$-law companding	Signal distortion, PAPR	Nonlinear LED response	Achieved 70% PAPR reduction with minimal BER increase (0.03)	PAPR reduction, BER
^8^ 2022	Hybrid VLM + GM precoding	PAPR, BER preservation	High PAPR, LED nonlinearity	Combined VLM & GM for better PAPR reduction (9.5 dB)	PAPR (dB), BER
^5^ 2023	Non-distorting precoding (DCT, DST, VLM, DHT)	PAPR, data rate preservation	High PAPR without side info	PAPR reduction: VLM (2.17 dB), DST (2.12 dB), DCT (1.46 dB), DHT (1.35 dB)	PAPR reduction (dB)
^6^ 2024	MPC + DPSO (Multi-point Constellation)	DPSO iterations, PAPR	LED nonlinearity, high PAPR	Improved PAPR reduction with increasing DPSO particles/iterations	PAPR reduction efficiency
^12^ 2024	VLM with modified O-OFDM (LACO/ASCO/FLIP/ACO/ADO/DCO-OFDM)	PAPR, data rate	High PAPR in multi-carrier systems	No side info required, maintained data rate	PAPR reduction, system throughput
^13^ 2024	WHT/DST + FDNC/TDNC noise cancellation	SNR, BER, PAPR	Noise and fading in ACO-OFDM	SNR improvement (14.64% WHT, 10.10% DST), PAPR reduction (6.76%−20.83%)	SNR (%), PAPR (%), BER
^14^ 2025	ML regressor (LPA)	SNR and BER	Fixed DC bias	DC bias Optimization BER improvement	SNR (dB), BER
^15^ 2025	Hybrid CNN-DNN	SNR and BER	LED nonlinearity	LED nonlinearity mitigation BER improvement	SNR (dB), BER

In this work, we developed a DCO-OFDM-based VLC system that enables real-time patient monitoring in biological healthcare applications. The proposed system adopts the selective mapping PR-coding (SLM PC) technique to greatly reduce PAPR, hence improving the monitoring signal’s quality, and transmission efficiency, in order to solve this constraint associated with high PAPR. We also employ four alternative equalization techniques: block-type, comb-type, ST, and blind CE to mitigate the effects of optical channel fading, which is especially significant in dynamic indoor environments with patient movement. The suggested solution shows that VLC can be a more efficient and superior substitute for RF systems in medical monitoring by overcoming these crucial obstacles. It provides a continuous patient data transmission solution that is safe, interference-free, and energy-efficient. The main contributions of this work are summarized as follows:

- A blind channel estimation is integrated with SLM-based PAPR reduction, specifically targeting a dynamic healthcare LiFi scenario.
- A comprehensive comparative analysis of channel estimation techniques under realistic patient positioning conditions is provided including LOS and NLOS cases.
- The trade-off between spectral efficiency and energy-to-noise ratio within this framework is analyzed.
- A significant PAPR reduction is shown through results with enhanced power efficiency, and improved overall system performance in clinical environments.

The structure of the remaining sections is as follows. Section "Multicarrier modulation based on LIFI system" demonstrates MCM, particularly DC-biased optical OFDM (DCO-OFDM) and Asymmetrically clipped optical OFDM (ACO-OFDM). Section “Equalizers” illustrates four types of equalizers, including block type, comb type, ST, and blind channel estimation (CE). Section "PAPR reduction and SLM precoding" introduces PAPR reduction algorithms; while Section "Proposed system and channel models" describes the proposed OFDM VLC system model and channel in detail. Section "Simulation results and discussion" discusses the experimental results along with Section “Computational complexity” which explains the computational complexity. Finally, Section "Conclusion and future work" provides a brief conclusion and future work.

## Multicarrier modulation based on LIFI system

For a VLC system, modulation technique is whether single-carrier modulation (SCM) of smaller data rates, such as pulse amplitude modulation (PAM), on-off keying, and other types of analog pulse modulation or MCM, such as optical OFDM (O-OFDM) for applications, that need higher data rates^12^. O-OFDM can offer enhanced communication capacity, and great data rates for optical LEDs through using wide light frequencies range^16^. According to the LED nonlinearity, BER, power efficiency, energy efficiency, and computational complexity, required for the VLC system, O-OFDM system is modified to explore several modified systems like FLIP-OFDM, ACO-OFDM, DCO-OFDM, ADO-OFDM, LACO-OFDM, unipolar OFDM (U-OFDM), and ASCO-OFDM^12^.

### DCO-OFDM

DCO-OFDM is considered among of the most standard modified O-OFDM MCM techniques, used for VLC systems. The transmitter starts with a mapper, which modulates the serial data according to the modulation type to obtain a number of complex symbols. The modulated symbols, that are in serial form are converted to a parallel form of *N*/2 columns, that represent half of the subcarriers number^12^. For transmitting only real data, Hermitian symmetry (H.S) is applied for the *N*/2 symbols, where they are flipped and conjugated to get additional *N*/2 symbols as follows^12^:

1 $$\begin{aligned} X(K)= X^{*}(n-K) \end{aligned}$$

Where *n* is the indices of subcarriers, located in the range $1\le K \le \frac{N}{2}- 1$. Currently, we obtain the modulated OFDM complex symbols are in the frequency domain as follows^17^:

2 $$\begin{aligned} X(K)= [X(0),X(1),...,X\bigg (\dfrac{N}{2}-1\bigg ),X\bigg (\dfrac{N}{2}\bigg ),..., X(N-1)] \end{aligned}$$

Inverse fast Fourier transform (IFFT) is applied with size *N* to translate them to the time domain *x*(*n*) as follows^13^:

3 $$\begin{aligned} x(n)= \frac{1}{\sqrt{N}}\sum _{k=0}^{N-1} X(K) e^{j\frac{2\pi K n}{N}},\qquad n=0,1,2,...,N-1 \end{aligned}$$

Then, serialize the symbols, and add dc bias $Bias_{DC}$, that is calculated as a function of expectation of the signal $E(x^{2}(n))$ as follows^17^:

4 $$\begin{aligned} Bias_{DC}=A \sqrt{E(x^{2}(n)}) \end{aligned}$$

Where *A* is a proportionality constant. After *DC* bias addition, the signal $x_{dc}(n)$ is expressed as follows^12^:

5 $$\begin{aligned} x_{dc}(n)=x(n)+Bias_{DC} \end{aligned}$$

The obtained samples contain positive and negative values; hence, their polar form are converted to unipolar through clipping them at level zero. A cyclic prefix $n_{CP}$ is included pre data transmission through the optical fading channel. At the receiver, reverse procedures are applied to reconstruct the original serial data^12^.

### ACO-OFDM

ACO-OFDM system offers a power-efficient replacement for the DCO-OFDM scheme with no need for *DC* bias. Unlike the DCO-OFDM technique, which incorporates a DC bias to guarantee all transmitted signals remain positive (a necessity for intensity modulation in optical systems), the ACO-OFDM technique accomplishes this using asymmetric clipping. In ACO-OFDM, the time-domain OFDM signal is clipped at zero, which means that all negative signal values are set to zero. Without adding a *DC* bias that consumes power, this process naturally produces a unipolar signal suitable for optical transmission. Importantly, since ACO-OFDM modulates data on just odd subcarriers and sets the even subcarriers to zero, this clipping process does not result in data loss. By ensuring that the additional clipping noise only affects the even subcarriers, which are devoid of any useful information, this design preserves the data’s integrity^13^.

ACO-OFDM is particularly useful in situations when power is limited, such as biomedical monitoring systems, because of its structure, which greatly increases power efficiency^18^. It is particularly well-suited for wearable or portable patient monitoring devices that rely on battery-powered VLC systems, where efficient power consumption is critical. For that system, the bit stream of the input signal is firstly mapped to number of complex symbols according to the modulation technique used, such 16-QAM or 64-QAM. The stream is then transformed from serial to parallel with *N*/2 columns^19^. For transmitting only real data, H.S is applied for the *N*/2 symbols, where they are flipped and conjugated to get additional *N*/2 symbols as previously explained in equation 1^12^. Serial data is parallelized, then applied to IFFT to get samples in the time domain as illustrated in equation 3. After that, the samples are converted back to serial and clipped at zero level with no information loss due to the symmetry around N/2 samples as expressed as follows^16^:

6 $$\begin{aligned} x\bigg (n+\frac{N}{2}\bigg )=-x(n), \qquad n=0,1,2,3,....,\frac{N}{2}-1 \end{aligned}$$

The clipped discrete samples $x_{clip}(n)$ are expressed as^16^:

7 $$\begin{aligned} x_{clip}(n)= \left\{ \begin{array}{l} x(n), \qquad \text {for } x(n)\le 0 \\ 0, \qquad \text {for } x(n) \le 0 \end{array} \right. \end{aligned}$$

To avoid the ISI, *CP* is included in each symbol header^20^. Therefore, the signal modulates the LED, whose light intensity is then sent through the optical fading channel. At the side of the receiver, photodetector (PD) can receive the optical signal to be detected and transformed to an electrical, followed by removing CP and a serial-to-parallel converter^21^. The discrete time symbols *y*(*n*) is translated in frequency domain *Y*(*k*) using fast Fourier transform (FFT) as follows^16^:

8 $$\begin{aligned} Y(K)= \sum _{k=0}^{N-1} y(n) e^{-j\frac{2\pi K n}{N}}, \qquad n=0,1,2,...,N-1 \end{aligned}$$

Then, the original symbols are detected by removing the symmetric samples added through H.S. at the transmitter, serializing, and demodulating them to reconstruct the original bit stream^12^.

## Equalizers

### Block type

**Fig. 1:**
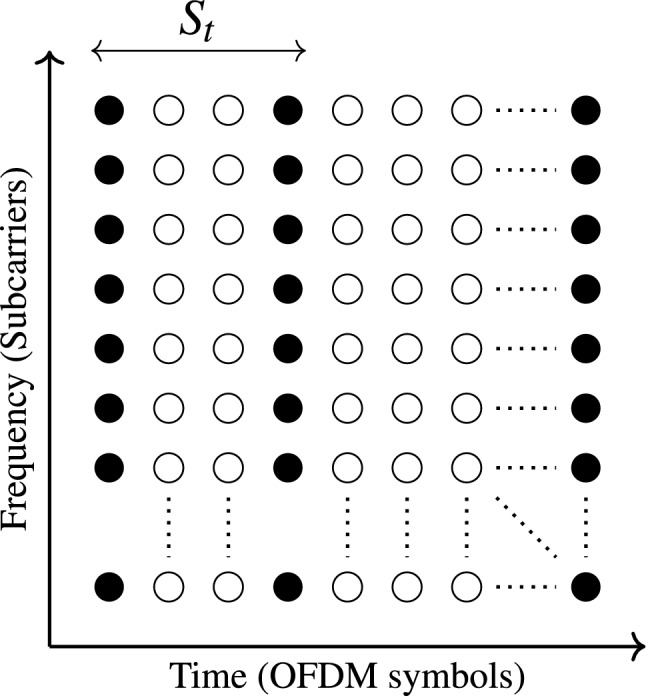
Pilot arrangement of block type CE of $S_{t}=2$.

Pilots are inserted into OFDM symbols with two basic methods, including block-type, and comb-type^22^. They are inserted after modulation at the side of the transmitter and removed pre the demodulation at the side of the receiver^23^. In block-type OFDM VLC system, the pilots are placed in all subcarriers in OFDM symbols, spaced by a specific pilot interval in the time domain ($S_{t}$) to be transmitted periodically as shown in Fig. 1. Block-type pilot insertion is used when the channel experiences slow fading and remains time-invariant over multiple symbols^24^.

The channel is estimated at the receiver by two methods, including linear minimum mean square error (LMMSE), and least squares (LS)^25^. LS estimation’ idea depends on reducing the weighted errors between the data and the developed model. The LS estimation for channel transfer function $H_{LS}$ is expressed as follows^26^:

9 $$\begin{aligned} H_{LS} =\dfrac{Y(K)}{X(K)},\qquad K=0,1,2,...,N-1 \end{aligned}$$

Where *X*(*k*) are transmitted symbols, and *Y*(*k*) are detected symbols at the receiver in frequency domain. Using LMMSE estimation, which is preferable compared to LS estimation for block-type (despite its higher complexity), the idea is based on using the channel frequency correlation, as expressed as follows^27^:

10 $$\begin{aligned} H_{LMMSE} =H_{LS} \ R_{HH_{LS}} \ R_{H_{LS}H_{LS}}^{-1} \end{aligned}$$

Where *H* is a linear combination of LS estimation, $R_{HH}$ is the auto-covariance matrix of *H*, and $R_{HH_{LS}}$ is the cross-covariance matrix between H and $H_{LS}$.

Decision feedback equalizers (DFEs) are employed at each subcarrier to estimate the channel inside the block in greater channel slow fading. This is carried out in two simple steps: first, use the prior channel response to estimate the transmitted signal $X_{e}(K)$ as follows:

11 $$\begin{aligned} X_{e}(K)=\frac{Y(K)}{H_{LMMSE}}, \qquad K=0,1,2,...,N-1 \end{aligned}$$

To update the channel response $H_{e}$, use the estimated transmitted signal in the manner described below:

12 $$\begin{aligned} H_{e}=\frac{Y(K)}{X_{e}(K)}, \qquad K=0,1,2,...,N-1 \end{aligned}$$

It has been noted that the DME concept is predicated on assuming a correct decision and then updating the channel. The equalization results in the predicted channel loss for fast fading channels. In that situation, it is necessary to balance the loss coming from the interpolation with that loss. Therefore, comb type equalizers are good fit for estimating fast fading channels.

### Comb type

**Fig. 2:**
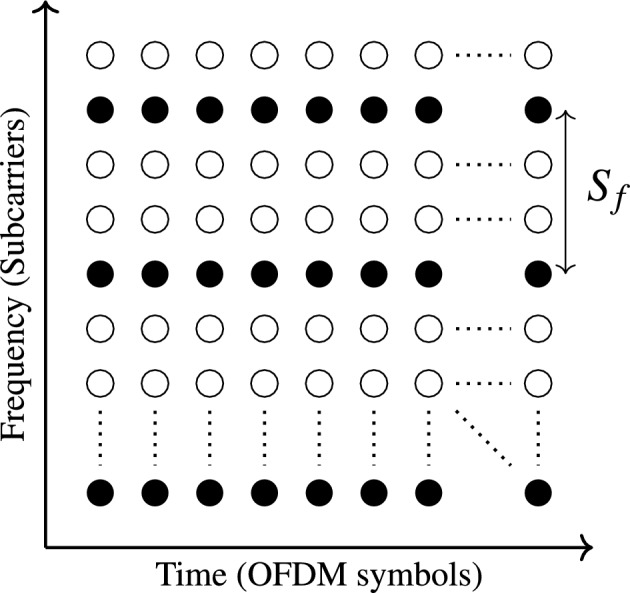
Pilot arrangement of comb type CE of $S_{f}=2$.

On the contrary, for comb-type equalizer, which is suitable for fast fading channel, the pilots are included in every OFDM symbol, periodically at a set of subcarriers with pilot spacing in the frequency domain ($S_{f}$) as shown in Fig. 2^28^. The pilots are inserted into the transmitted signal *X*(*K*) with a uniform manner as follows:

13 $$\begin{aligned} X(K)= X(n_{1} S_{f}+n_{2}) = \left\{ \begin{array}{l} pilot, \qquad \text {when } n_{2}= 0 \\ Data, \qquad \text {when } n_{2}= 1,2,...,S_{f}-1 \end{array} \right. \end{aligned}$$

Where ($n_{1} = 1,2,....N/s_{f}$). LS estimation is used to estimate the channel response of pilot subcarriers $H_{e_{comb}}$ as follows:

14 $$\begin{aligned} H_{e_{comb}}=\frac{Y_{p}(K)}{X_{p}(K)} \qquad K= 0,1,......,(N/s_{f})-1 \end{aligned}$$

Where $X_{p}(K)$ and $Y_{p}(K)$ are transmitted and received pilot subcarriers, respectively. Because LS estimation is vulnerable to noise and intercarrier interference, it is not recommended. The LMS estimator, inspite of its complexity, is based on estimating the first pilot symbol value using LMS filter (one-tap) at every pilot subcarrier, and calculating the following subsequent values based on previous and current estimations^28^. Accordingly, using the channel determined at the pilot subcarrier, the interpolation method is used to estimate the channel of data subcarriers *N*. For correct channel estimation, $S_{f}$ is set according to the following equation^27^:

15 $$\begin{aligned} S_{f} \le \frac{1}{\sigma _{max}} \end{aligned}$$

Where $\sigma _{max}$ is the maximum delay spread, caused by the channel^29^. Using first order linear interpolation is preferred, Channel response at data subcarriers $H_{e_{comb}}(K)$ is evaluated as follows:

16 $$\begin{aligned} H_{e_{comb}}(K)= H_{e_{comb}}(n_{1} S_{f}+n_{2})=\frac{1}{S_{f}}(H_{e_{comb}}(n_{1}+1)- H_{e_{comb}}(n_{1}))+H_{e_{comb}}(n_{1}),\qquad n_{2}= 0,2,...,S_{f} \end{aligned}$$

Where pilots’ channel response is modified to include zeros. The original data is then allowed to pass while maintaining the lowest mean square error (MSE) between the interpolated data and their ideal values by using a finite impulse response filter (FIR).

### Superimposed training (ST)

ST channel estimation (CE) is based on superimposing a non-random, low power pilot sequence onto the data sequence before the modulation step at the transmitter. To explain how a known pilot sequence $C(K)=(1+\frac{n_{sym}}{2})e^\frac{j2Kn_{sym}}{N}$ is algebraically added to the unknown broadcast signal *X*(*K*), the following formula is applied:

17 $$\begin{aligned} X_{imp}(K)=X(K)+C(K) \end{aligned}$$

Where $X_{imp}(K)$ is the superimposed transmitted signal, which is passed through all stages of the proposed DCO-OFDM VLC system. At the receiver, the pilot is estimated for CE and data equalization. Using a frequency window of size *SM*, the channel pilot response $C_{e}(K)$is estimated through averaging each SM block as follows^30^:

18 $$\begin{aligned} C_{e}(K)=\frac{\sum _{i~ SM}^{(i+1) SM} Y(K)}{SM} \qquad i= 0,1,......,n_{sym}/SM \end{aligned}$$

By using the opposite of the pilot approach at the transmitter, the data subcarrier’s received response $Y_{d}(K)$ is approximated as follows:

19 $$\begin{aligned} Y_{d}(K)=Y(K)-C_{e}(K) (1+\frac{n_{sym}}{2})e^\frac{j2Kn_{sym}}{N} \end{aligned}$$

With knowledge of the estimated channel and received signal, the original signal $X_{e}(K)$ is estimated as a function of QAM symbol power $S_{alpha}$ as follows:

20 $$\begin{aligned} X_{e}(K)=\frac{Y_{d}(K) \sqrt{\dfrac{S_{alpha}}{1-S_{alpha}}}}{\sqrt{2} ~ C_{e}(K)} \end{aligned}$$

### Blind CE

Blind CE is based on using a maximum likelihood estimation algorithm, where the received signal ($Y_{K}$) for *K* subcarriers is expressed as a function of $\alpha$ (channel impulse response), and $\theta$ (normalization factor), dependent on the distance from the patient, the zenith angle of its position, and ambient light factor, it is expressed as follows^22^:

21 $$\begin{aligned} Y_{K}=X_{K}\ \theta \ \alpha _{K}+N_{K} \end{aligned}$$

Where $X_{K}$ is the transmitted biomedical signal, and $N_{K}$ is the noise added to the receiver. Let $\beta _{K}=X_{K} \theta$, we can use the maximum likelihood estimation to get $\beta _{K}$ at the maximum value of $Y_{K}$ as follows^22^:

22 $$\begin{aligned} \beta ^{'}_{K}=\frac{Y_{K}}{\alpha _{K}} \end{aligned}$$

Then, the law of the large numbers is applied on $|\beta _{K}|=|X_{K} \theta |$ and the estimated $|\beta ^{'}_{K}|$ is used to obtain $\theta$ as follows^22^:

23 $$\begin{aligned} \theta = \dfrac{\lim _{N\rightarrow \infty } \frac{1}{N}\sum _{K=1}^{N} |\beta ^{'}_{K I}|+|\beta ^{'}_{K Q}|}{E\big (|X_{I}|+|X_{Q}|\big )} \end{aligned}$$

Where $X_{I}$ and $X_{Q}$ correspond to the real and imaginary random values of the transmitted signal, which depend on the type of modulation. By applying Equations 22 and 23, $X^{'}_{K}$ is estimated to be $X^{'}_{K}=\frac{\beta ^{'}_{K}}{\theta }$ as follows^22^:

24 $$\begin{aligned} X^{'}_{K}= \dfrac{N Y_{K} E\big (|X_{I}|+|X_{Q}|\big )}{\alpha _{K} \sum _{K=1}^{N} \frac{\big (|Y_{K I}|+|Y_{K Q}|\big )}{\alpha _{K}}} \end{aligned}$$

## PAPR reduction and SLM precoding

### PAPR

In VLC systems, the PAPR is a fundamental performance parameter used to assess the efficiency and dependability of the transmitted signal. The transmitted signal peak (maximum) instantaneous power to its average power over time ratio is known as PAPR. It serves as an indication of the LED dynamic range, that is required by the optical transmitter, such as LEDs, that are restricted in their linear operating region^31^. Mathematically, PAPR is expressed as:

25 $$\begin{aligned} PAPA=\dfrac{|x(n)|^{2}_{max}}{E(|x(n)|^{2})} \end{aligned}$$

Where *E*(.) is the statistical expectation operator, that is employed to determine the average power of the optical signal, transmitted. A high PAPR means that the signal exhibits strong power fluctuations, that causes various concerns, such as nonlinear distortion, poor power efficiency, and higher complexity for the optical source and driver circuits’ design^32^. In VLC systems used for healthcare patient monitoring applications, where consistent and dependable data delivery is essential, these impacts are especially important. Thus, reducing PAPR is essential to ensure efficient power use, extend the life of optical transmitters, and preserve signal integrity in the dynamic conditions frequently found in healthcare settings^33^..

In VLC systems, particularly those that use MCM techniques like OFDM, PAPR reduction is essential. The transmitted signal in MCM systems is made up of many subcarriers that can align constructively at particular times to produce simultaneous maximum amplitudes^5^. This phenomenon leads to a high PAPR due to the nonlinear response of optical transmitters such as LEDs, which can cause signal distortion and impair system performance^5^. This issue is often resolved and the effectiveness of PAPR reduction techniques evaluated using the Complementary Cumulative Distribution Function (*CCDF*). The *CCDF* calculates the likelihood that a transmitted signal’s PAPR metric will surpass a predetermined threshold value $PAPR_{0}$. Mathematically, it is defined as^12^:

26 $$\begin{aligned} CCDF=Prob(PAPR>PAPR_{0})=1-(1-e^{-PAPR_{0}})^{N} \end{aligned}$$

### Precoding PAPR reduction techniques

Precoding techniques (PCTs) are widely used by MCM-based VLC systems to effectively address the issue of high PAPR^34^. Numerous advanced precoding techniques exist, including VLM, WHT, DST, DCT, and SLM. The fundamental idea behind these techniques is to provide a uniform distribution of signal power by rearranging the energy of the transmitted symbols in both the temporal and frequency domains^5^.. This redesign reduces the PAPR without appreciably increasing system complexity or data loss by reducing the occurrence of strong peaks in the composite OFDM signal^5^.

essentially, a precoding matrix $M_{pc}$ of size $N\times N$ appropriate for the chosen technique is applied at the transmitter, especially before the IFFT stage^13^. Specifically, the data symbols to be transmitted are multiplied by the precoding matrix, which redistributes the symbols’ energy across multiple subcarriers^13^.

27 $$\begin{aligned} M_{pc}= \begin{pmatrix} P_{00} & P_{01} & \dots & P_{0(N-1)} \\ P_{10} & P_{11} & \dots & P_{1(N-1)} \\ P_{20} & P_{21} & \dots & P_{2(N-1)} \\ \vdots & \vdots & \ddots & \vdots \\ P_{(N-1)0} & P_{(N-1)1} & \dots & P_{(N-1)(N-1)} \end{pmatrix} \end{aligned}$$

This strategic distribution ensures that the power is spread more evenly over the transmission bandwidth, effectively lowering the probability of peak signal power events^35^. By integrating these precoding strategies into the system, not only is the PAPR significantly reduced, but also the power amplifier efficiency is enhanced, and the optical transmitters linear operation is better maintained which are critical requirements for reliable and energy-efficient VLC-based patient-monitoring applications^36^.

### SLM

**Fig. 3:**
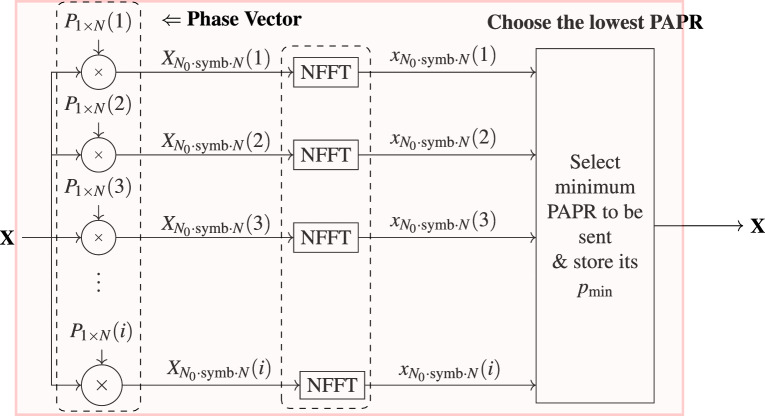
Composition of SLM transmitter and receiver.

SLM is one of the most widely used PCTs in OFDM-based VLC systems for PAPR reduction purpose. Its circuits at the transmitter and receiver is shown in Fig. 3. Its operational principle is centered on generating multiple candidate versions of the same OFDM data block, where each version is multiplied by a different, carefully designed phase rotation sequence before IFFT^37^, as expressed below:

28 $$\begin{aligned} P(i)=[P_{0}(i),P_{1}(i),P_{2}(i), \dots ,P_{N-1}(i)] \end{aligned}$$

where *i* is the iterations’ number, and $P_{j}(i)$ are complex numbers of $e^{j\theta }$. These phase vectors are created to ensure that the original data content remains preserved, meaning that no information loss occurs despite the phase alterations^37^. Following the generation of these many candidate signals, each candidate is evaluated to determine its PAPR value. The candidate with the lowest PAPR is then chosen by the system for actual transmission^38^.For the receiver to accurately reconstruct the original broadcast signal, side information, or the index of the chosen phase sequence, must be sent with the data^38^. This selection process greatly reduces the possibility of large instantaneous power peaks, which may otherwise create severe nonlinear distortion in VLC systems due to the limited linear range of optical sources like LEDs^38^.

By reducing these occurrences, SLM improves the VLC communication connection’s dependability and robustness, maximizes overall power efficiency, and lessens the load on the optical transmitter^7^. Because real-time health data tracking requires consistent, low-distortion data transmission, these advantages are particularly crucial for patient monitoring applications. It is crucial to remember that while SLM significantly improves PAPR performance, it also increases computing demands at the transmitter and requires sending side information (phase sequence) to enable accurate demodulation at the receiver^39^.

## Proposed system and channel models

### Model of the proposed system

**Fig. 4:**
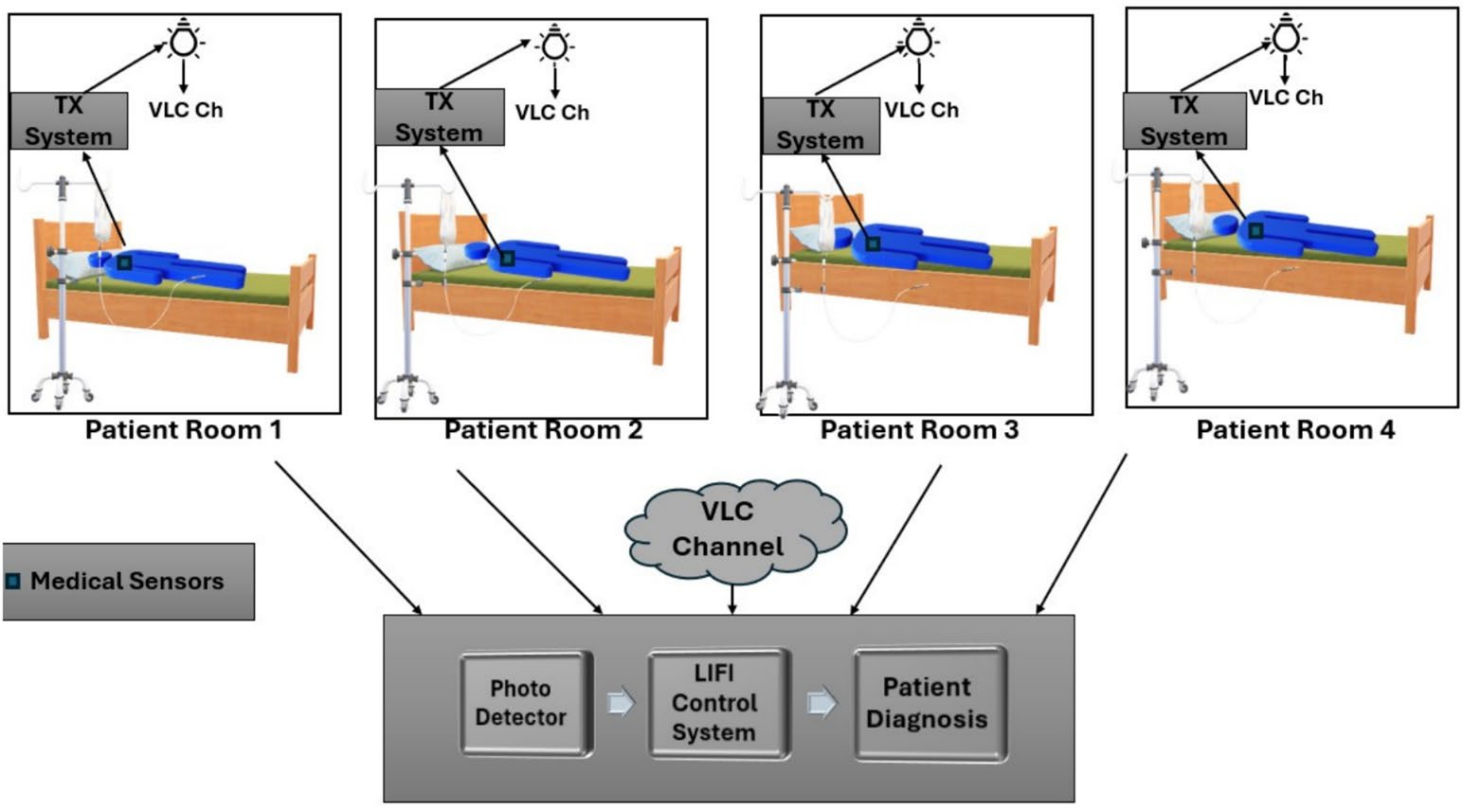
VLC Healthcare Monitoring System.

The proposed DCO-OFDM-based VLC system is structured into three fundamental sections: the first transmitter block, the VLC optical fading channel, and the final receiver block. It is used for real time patient monitoring as shown in Fig. 4. This technology was created especially to function well in biomedical healthcare settings where patients are being monitored. The transmitter portion generates and modulates the DCO-OFDM signals suitable for optical transmission. These signals are then transmitted through a VLC optical channel, which is made to adjust for the different lighting conditions, body posture, and motion that are typical in a patient-care environment.

**Fig. 5:**
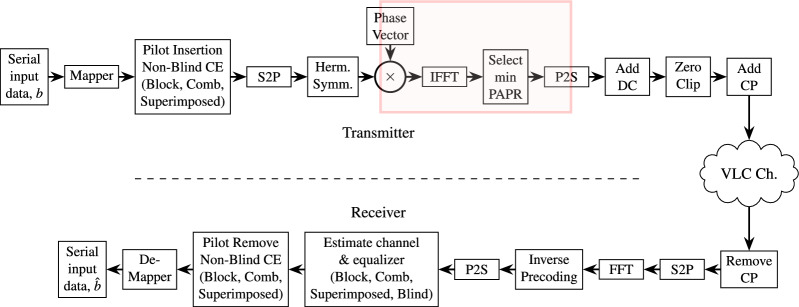
Block diagram of the proposed system for patient monitoring.

To mitigate the effects of multipath fading and signal distortion brought on by the VLC channel, the system employs four different types of channel equalizers at the receiver end. These equalization techniques are thoroughly explained in the system’s block diagram in Fig. 5, which highlights their various roles in guaranteeing precise and reliable signal reception in a variety of patient circumstances.

To address the important PAPR issue in OFDM-based systems, we employed selective mapping (SLM), one of the best precoding techniques. The intended DCO-OFDM-VLC system’s transmitter component made selective use of this technique. Specifically, the SLM precoding block was followed by the Inverse Fast Fourier Transform (IFFT) stage. The system can produce numerous candidate OFDM signals with different phase sequences, each of which represents the same data. The power amplifier’s efficiency is significantly increased and signal distortion is reduced when the sequence with the lowest PAPR among these is selected for transmission. The Fast Fourier Transform (FFT) block is immediately followed at the receiver end by an inverse SLM decoding process. By reversing the phase sequence used during precoding, this guarantees accurate reconstruction of the original data delivered. By adding SLM into the system, we realized a considerable reduction in PAPR, resulting to increased signal quality, lower bit error rates, and improved system reliability vital elements for monitoring patients in real time in delicate biomedical settings.

### Channel model

Accurate channel modeling is crucial for designing effective equalization and estimation algorithms. This Section presents an enhanced channel model for VLC systems in patient-monitoring environments. The model extends conventional optical channel impulse response formulations by incorporating frequency-domain representations suitable for Orthogonal Frequency Division Multiplexing systems. The proposed formulation accounts for multipath propagation, geometric and photometric parameters, and normalization factors specific to healthcare settings. Simulation results demonstrate the model’s accuracy in capturing channel variations due to patient movement and environmental dynamics. Consider an OFDM-based VLC system with *N* subcarriers. The transmitted signal in the frequency domain is denoted as *X*[*k*] for $k = 0, 1, \dots , N-1$. After passing through the optical channel, the received signal at the *j*-th receiver can be expressed as^22^:

29 $$\begin{aligned} Y_j[k] = X[k] H_j[k] + N_j[k], \end{aligned}$$

where $N_j[k]$ is additive white Gaussian noise with zero mean and variance $\sigma ^2$, and $H_j[k]$ is the frequency response of the channel between the transmitter and the *j*-th receiver.

The optical channel impulse response (CIR) for VLC systems in an indoor environment can be modeled as a sum of attenuated and delayed components corresponding to line-of-sight (LOS) and non-line-of-sight (NLOS) paths^40^:

30 $$\begin{aligned} h_j(t) = \sum _{i=1}^{N_r} D_i \cdot \delta (t - \tau _i), \end{aligned}$$

where:

- $N_r$ is the total number of propagation paths,
- $D_i$ is the gain of the *i*-th path, which depends on geometric and photometric parameters,
- $\tau _i$ is the delay of the *i*-th path,
- $\delta (\cdot )$ is the Dirac delta function.

The path gain $D_i$ for an optical link is given by^40,41^:

31 $$\begin{aligned} D_i = {\left\{ \begin{array}{ll} \frac{(m+1)A}{2\pi d_i^2} \cos ^m(\phi _i) \cos (\psi _i) \text {rect}\left( \frac{\psi _i}{\Psi _c}\right) , & \text {LOS},\\ \frac{\rho A}{\pi d_i^2} \cos (\phi _i) \cos (\psi _i) \text {rect}\left( \frac{\psi _i}{\Psi _c}\right) , & \text {NLOS}, \end{array}\right. } \end{aligned}$$

where:

- $m = -\ln 2 / \ln (\cos \Phi _{1/2})$ is the Lambertian order,
- *A* is the detector area,
- $d_i$ is the distance traveled by the *i*-th path,
- $\phi _i$ and $\psi _i$ are the irradiance and incidence angles, respectively,
- $\Psi _c$ is the field of view of the receiver,
- $\rho$ is the reflection coefficient of the surface (for NLOS).

The frequency response $H_j[k]$ is obtained by taking the Discrete Fourier Transform (DFT) of the sampled CIR:

32 $$\begin{aligned} H_j[k] = \sum _{n=0}^{N-1} h_j[n] e^{-j 2\pi k n / N}, \end{aligned}$$

where $h_j[n] = h_j(n T_s)$ is the sampled version of the CIR with sampling period $T_s$.

In a dynamic patient-monitoring environment, the channel may vary due to patient movement, changes in ambient light, and equipment mobility. To account for this, we introduce a time-varying normalization factor $\theta _j[n]$ that scales the received signal power based on the distance and orientation between the patient (wearable device) and the receiver. The normalized received signal in the frequency domain becomes:

33 $$\begin{aligned} \tilde{Y}_j[k] = \theta _j[k] *\left( X[k] H_j[k] \right) + N_j[k], \end{aligned}$$

where $*$ denotes convolution and $\theta _j[k]$ is the DFT of $\theta _j[n]$.

The patient-monitoring scenario is assumed to take place in a room of dimensions $3\,\text {m} \times 4\,\text {m} \times 2.5\,\text {m}$. The receiver is placed on the ceiling, and the patient wears a wearable device that acts as a transmitter. The following parameters are considered:

- Distance between transmitter and receiver: $d \in [0.5, 3]\,\text {m}$,
- Zenith angle $\psi$: varies with patient posture (lying, sitting, standing),
- Ambient light intensity: modeled as a DC bias plus low-frequency noise,
- Reflectivity of walls and medical equipment: $\rho \approx 0.6$–0.8.

These factors are incorporated into the path gain model in (31) and the normalization factor $\theta _j[n]$.

The following system parameters are used for evaluation:

**Table 2:** Simulation Parameters.

Parameter	Value
Room dimensions	$3\,\text {m} \times 4\,\text {m} \times 2.5\,\text {m}$
Number of data bits	230400
Number of symbols	600
Cyclic prefix $n_{cp}$	4
Guard	8
Number of subcarriers *N*	64
Active subcarriers $n_{sc}$	48
Modulation schemes *M*	QPSK and 16-QAM
Bandwidth	20 MHz
Sampling rate $f_s$	44.1 kHz
PS for block type $S_{t}$	2
PS for comb type $S_{f}$	3
DC Bias	0.5
Phase sequence *U*	3 real-3complex
LED transmission power	1
Photo detector sensitivity	1
Guard interval length	256 samples
Channel model	Multipath optical fading
Noise model	AWGN + ambient light noise

## Simulation results and discussion

This study’s simulation results rely on a 230,400-bit serial data set that was generated at random with the goal of simulating real-world biomedical signals that are generally collected via wearable medical sensors. A variety of biological metrics, including body temperature readings, heart rate measurements, and electrocardiogram (ECG) waveforms, are represented in this simulated dataset. The system’s performance is evaluated under conditions that closely mimic the constant and variable nature of medical data transmission in real medical monitoring applications by incorporating a broad range of biological signals. This method guarantees that the VLC-based OFDM system is thoroughly assessed for robustness, dependability, and effectiveness in transmitting vital medical data.

This section presents and analyzes the MATLAB simulation results for the DCO-OFDM based VLC healthcare monitoring system, both with and without the SLM precoding technique. The simulation results concentrate on the critical measures for performance required for evaluating system efficacy, including the BER, Spectral Efficiency, and PAPR.

To accurately assess system performance under real-world conditions, four types of equalizers namely, block-type, comb-type, superimposed, and blind CE were implemented at the receiver. These equalizers were used to compensate for channel impairments and to equalize the received biometric data across three distinct patient positioning scenarios, representing common variations in a hospital or homecare environment (e.g., sitting, lying down, or moving).

**Fig. 6:**
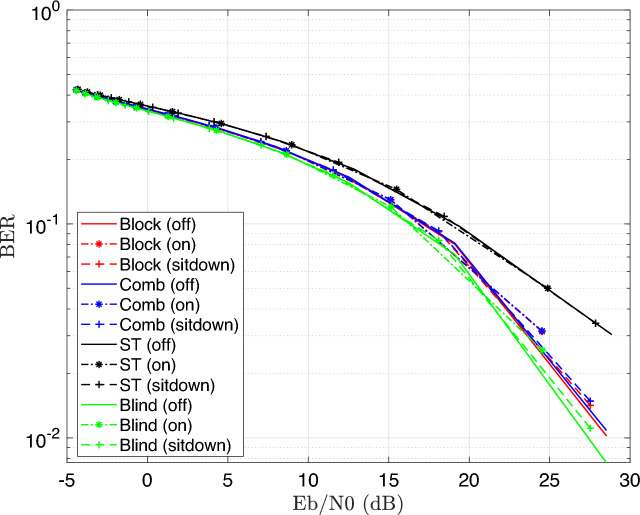
BER performance of the DCO-OFDM-based VLC healthchare monitoring system using four equalization techniques across three patient positioning scenarios (16QAM).

Figure 6 illustrates the BER performance with SNR increase for a 16-QAM modulated OFDM VLC system. The result shows that BER gets better as SNR rises, and highlights the effectiveness of the SLM technique in enhancing signal robustness and reducing PAPR, particularly under challenging channel conditions caused by patient mobility and varying transmission distances. It demonstrates that the performance differences among the three patient positioning scenarios such as light off, light on, or patient sitdown are minimal. This is mainly due to the simulated patient room model’s small size, which causes only slight differences in channel properties (such as path loss and multipath effects) between various patient positions; consequently, the system’s performance is largely unaffected by the patient’s location within the area, designed for simulation.

In addition, Fig. 6 illustrates the higher performance of the blind equalizer over the other equalization strategies. The increased accuracy in channel normalization, which is accomplished by using the Maximum Likelihood Estimation (MLE) approach, is primarily responsible for this improved performance. In situations where pilot-based estimation may be restricted or impractical, the MLE technique makes it possible for the blind equalizer to estimate the channel more precisely without the need for known pilot symbols.

**Fig. 7:**
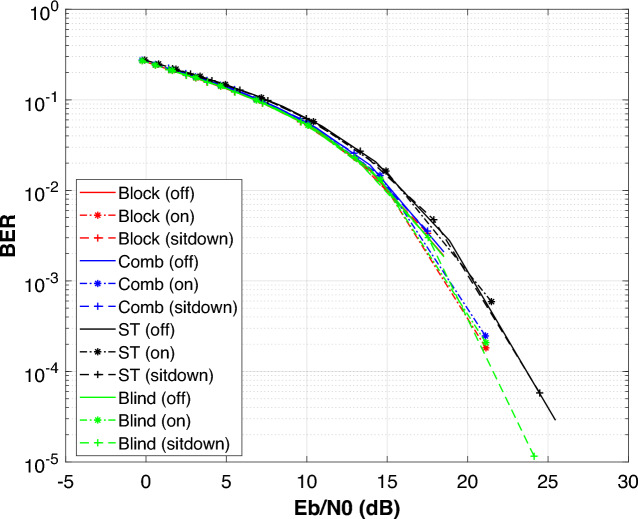
BER Estimation of the DCO-OFDM-based VLC healthchare monitoring system using four equalization techniques across three patient positioning scenarios (4QAM).

The Energy-to-Noise ratio is used in optical intensity modulation/direct detection (IM/DD) systems, as the signal constrained to non-negative optical power (DC valued, amplitude-only signals). Therefore, it directly relates the average optical power to noise and therefore a more appropriate and meaningful metric than electrical SNR for performance analysis in VLC systems. Similarity, using 4-QAM modulated OFDM VLC system, the simulation is re-executed as shown in Fig. 7.

**Fig. 8:**
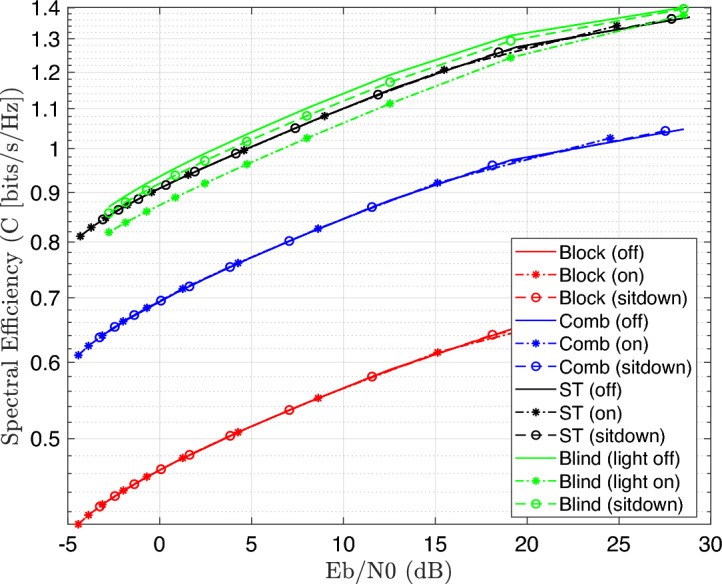
Spectral effeciency Vs. SNR performance of the DCO-OFDM-based VLC healthchare monitoring system using four equalization techniques across three patient positioning scenarios (16-QAM).

Figure 7 illustrates the BER performance with SNR increase for 4-QAM modulated OFDM VLC system. The results introduce a better improvement in BER, which reached to $10^{-5}$ at SNR 25dB as compared to 16-QAM system, which achieved $10^{-2}$ at the same SNR. Moreover, the spectral efficiency was computed for 16-QAM modulated OFDM VLC system at different SNR values as explained in Fig. 8 as illustrated in the following equation:

34 $$\begin{aligned} \gamma =\frac{log_{2}M+n_{sc}/2}{N+n_{cp}}(1-BER) \end{aligned}$$

**Fig. 9:**
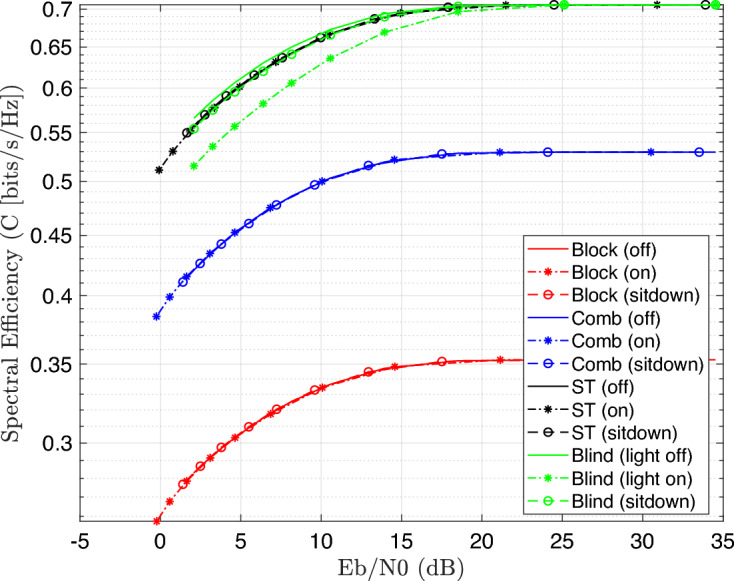
Spectral effeciency Vs. SNR performance of the DCO-OFDM-based VLC healthchare monitoring system using four equalization techniques across three patient positioning scenarios (4-QAM).

Where *M* is the QAM order, $n_{sc}$ is the number of active subcarriers, *N* is Fourier size, $n_{cp}$ is the cyclic prefix, and BER is the bit error rate. The results indicate that spectral efficiency improves with increasing SNR. Notably, the blind equalizer regularly achieves superior spectral efficiency, thanks to its robust channel estimation abilities without the requirement for pilot overhead, which makes it particularly ideal for higher-mobility or low-pilot circumstances in health care applications. Similarity, the spectal effeciency is re-computed at 4-QAM modulated system as explained in Fig. 9.

**Fig. 10:**
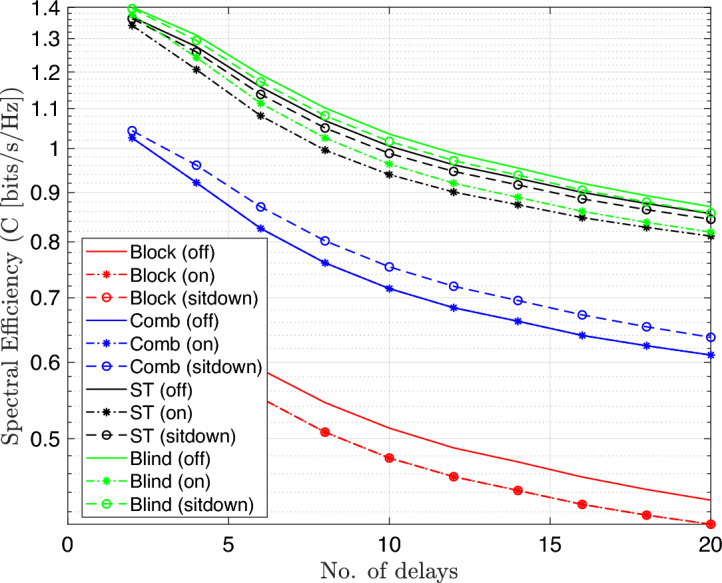
Spectral efficiency Vs. no.delays performance of the DCO-OFDM-based VLC healthcare monitoring system using four equalization techniques across three patient positioning scenarios(16-QAM).

The results explain that the spectral efficiency is reduced by nearly 50$\%$ as compared to 16-QAM system because the number of bits per each symbol decreases to half. Moreover, the spectral efficiency was computed for 16-QAM modulated OFDM VLC system at different number of multipath delays as explained in Fig. 10.

**Fig. 11:**
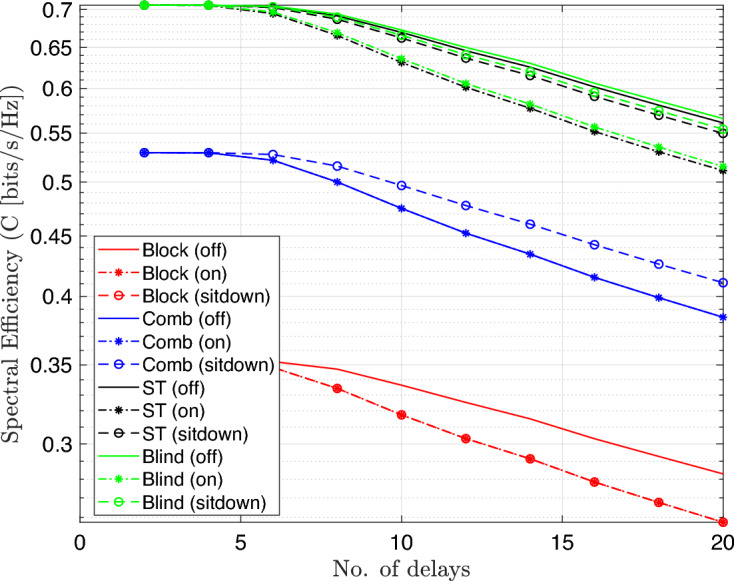
Spectral effeciency Vs. no.delays performance of the DCO-OFDM-based VLC healthchare monitoring system using four equalization techniques across three patient positioning scenarios(4-QAM).

The results show the impact of channel multipath delay on spectral efficiency for modulation scheme 16-QAM, where it decreases as the multipath delays’ number increases due to greater inter-symbol interference. Using 4-QAM system, the simulation is repeated as shown in Fig. 11. The results indicate that the spectral efficiency decreases to nrealy 50 $\%$ as compared to 16-QAM at the same conditions. In both sets of simulations, the system is tested under three different patient positioning scenarios and utilizes four distinct equalization techniques: block-type, comb-type, superimposed, and blind equalizers. The aim is to assess how each equalizer adapts to dynamic changes in the channel environment, which may arise from patient movement or increased channel dispersion.

**Fig. 12:**
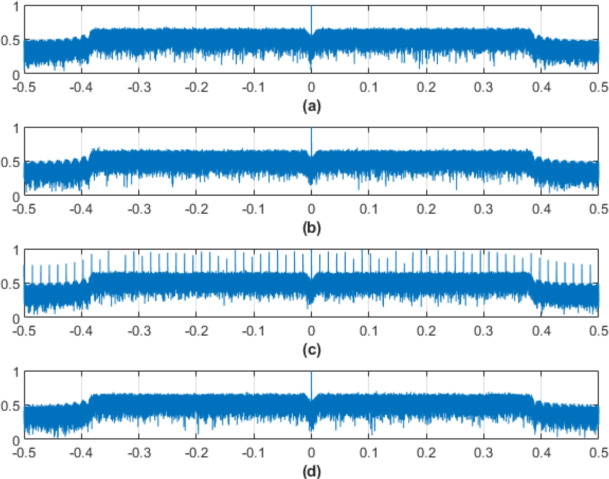
Frequency-domain spectrum of the transmitted signals of the DCO-OFDM-based VLC healthcare monitoring system using (a) Block Type equalizer, (b) Comb Type, (c) ST, and Blind CE.

The frequency-domain spectra of the transmitted signals before the IFFT is applied for each of the four equalization approaches as shown in Fig. 12. The modulated data symbols applied to the OFDM subcarriers before are translated into the time domain for optical transmission correspond to this spectrum.

This level of signal spectrum analysis clarifies how each equalization method impacts the distribution of signal strength among the subcarriers and the uniformity of spectrum occupancy. Frequently, a dense and well-balanced spectral distribution shows reduced susceptibility to frequency-selective fading and effective bandwidth utilization. The spectrum characteristics depicted in the figure also illustrate variances in symbol energy density that might influence system performance after IFFT, especially with regard to PAPR and channel robustness. This pre-IFFT view serves as a basis for assessing the effects of SLM and other precoding methods used later in the transmission chain and is crucial for comprehending the initial conditions from which the time-domain signal is produced.

**Fig. 13:**
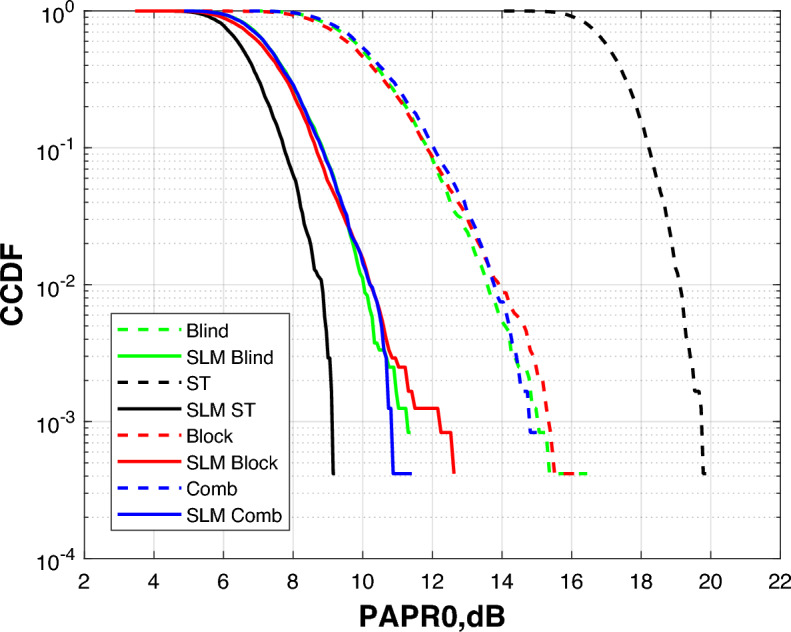
PAPR estimation of the DCO-OFDM-based VLC healthchare monitoring system using four equalization techniques.

The superior performance of Blind channel estimation in our dynamic scenarios in terms of BER and spectral efficiency is attributed to its ability to track channel variations without dedicated pilot symbols. This makes it more robust to the fading and multipath effects induced by patient movement (NLOS conditions), whereas pilot-based methods (block, comb, ST) suffer from overhead and potential pilot contamination in such dynamic environments. From a PAPR perspective, however, the superimposed training equalizer shows slightly better performance as explained in Fig. 13.

For high PAPR issue solution in OFDM-based VLC systems, SLM technique is employed. SLM reduces PAPR effectively through producing several statistically independent versions of the same OFDM signal, each modified with a distinct phase sequence. One of the benefits of this approach is the reduction of autocorrelation between adjacent symbols, which helps to suppress power peaks and ensures a more uniform signal envelope in the time domain. Figure 13 shows how SLM affects the DCO-OFDM VLC healthcare monitoring system’s PAPR performance as assessed by four alternative equalization methods. A direct comparison of SLM’s efficacy is possible because the findings are displayed both with and without applying it. SLM considerably reduces the PAPR for all equalization types, as shown in Fig. 13.

In addition to improving transmission reliability and power efficiency—two crucial aspects in biomedical monitoring environments—this modification additionally reduces signal distortion brought on by the nonlinear properties of optical components (like LEDs). Blind equalizer achieved an effective power distribution and adaptive channel estimation, it is combined with SLM to exhibit the best PAPR decrease among the equalizers.

**Fig. 14:**
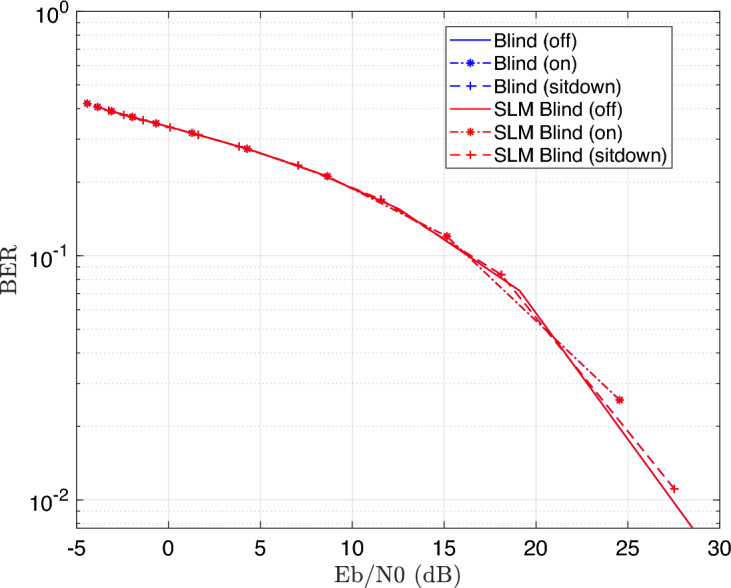
BER estimation of the DCO-OFDM-based VLC healthchare monitoring system employing blind CE, with and without the application of the SLM technique.

These results offer a comprehensive comparison of how each equalizer performs under different channel conditions and how the integration of SLM influences system reliability. The results show that using SLM improves BER significantly for all equalizers. The ability of SLM to reduce the PAPR, which reduces the nonlinear distortion brought on by the VLC transmitter components, is the primary source of this improvement. As a result, the received signal deteriorates less, improving the entire error performance. Figure 14 illustrates the BER performance of the proposed DCO-OFDM based VLC healthcare monitoring framework, evaluated across blind CE equalizer technique with and without the application of the SLM precoding technique.

**Fig. 15:**
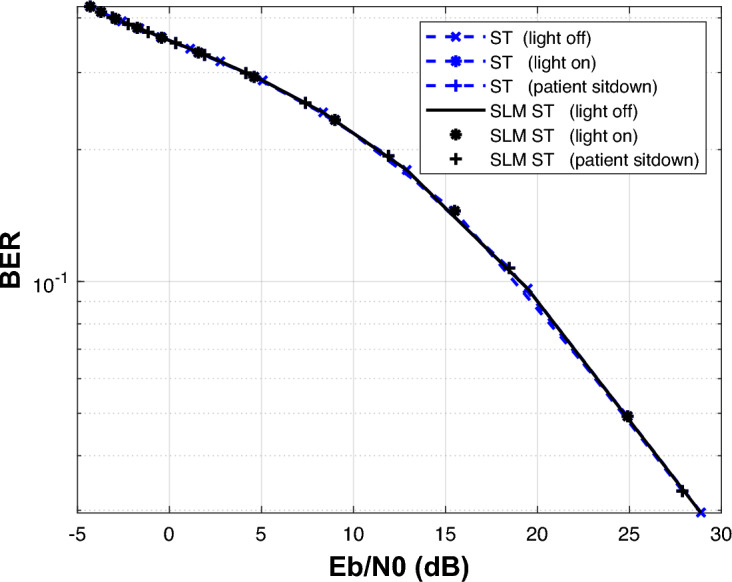
BER estimation of the DCO-OFDM-based VLC healthchare monitoring system employing ST CE, with and without the application of the SLM technique.

The results illustrate that SLM precoding technique can preserve BER without degredation. Similarty, The simulated is repeated for superimposed equalizer with and without the application of the SLM precoding technique as shown Fig. 15.

**Fig. 16:**
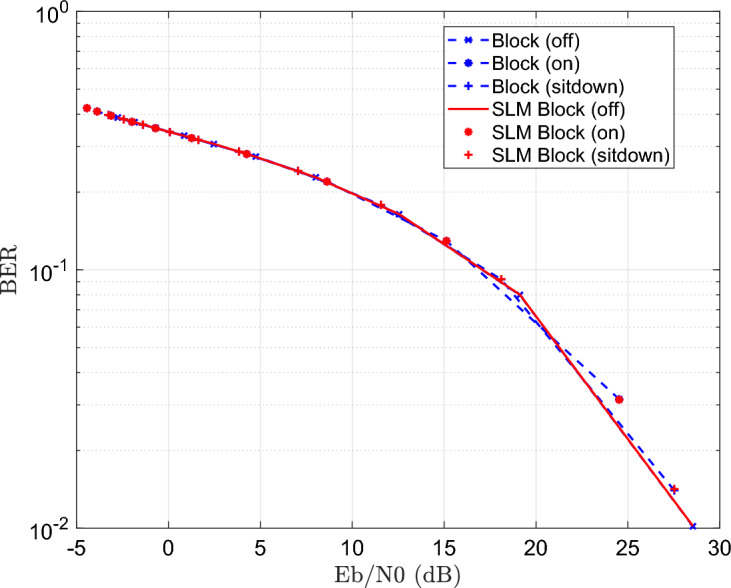
BER estimation of the DCO-OFDM-based VLC healthchare monitoring system employing block-type CE, with and without the application of the SLM technique.

The simulation is repeated for block type equalizer with and without the application of the SLM precoding technique as shown Fig. 16.

**Fig. 17:**
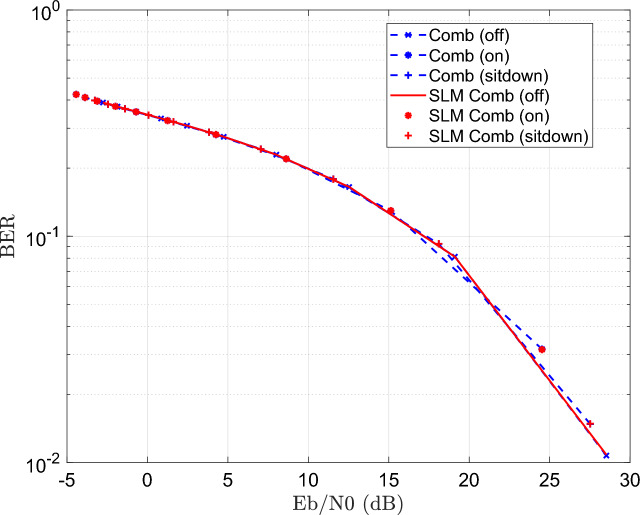
BER estimation of the DCO-OFDM-based VLC healthchare monitoring system employing comb-type CE, with and without the application of the SLM technique.

The results illustrate BER performance without degradation, and improvement as compared to ST equalizer. For the comb equalizer, the simulation is repeated as explained in Fig. 17.

The results explains how SLM preserve the BER performance with PAPR improvement at the same time.

The blind equalizer consistently produces the best BER values among the equalization techniques, especially when paired with SLM. While the proposed method achieves a significant reduction in BER for blind equalizer as compared to the other equalizers shown in Figs 15, 16 and 17. But superimposed training equalizer Slightly superior related to PAPR after applying SLM technique. This behavior is expected, as PAPR reduction techniques may introduce distortion that impacts some equalizers. This is ascribed to its strong channel estimating capabilities and adaptive normalization, which are successful even without pilot symbols. These findings underscore the need of concurrently enhancing PAPR reduction and equalization to provide reliable and accurate transmission of biological data in indoor healthcare monitoring conditions. Apart from the earlier simulations, the SLM approach was implemented using real-valued phase vectors in all previous assessments. An further simulation employing complex-valued phase vectors was carried out to further examine how phase vector design affects system performance. These complex vectors, often of the form $e^{j\Theta }$, add both amplitude and phase changes while retaining the signal’s overall energy.

**Fig. 18:**
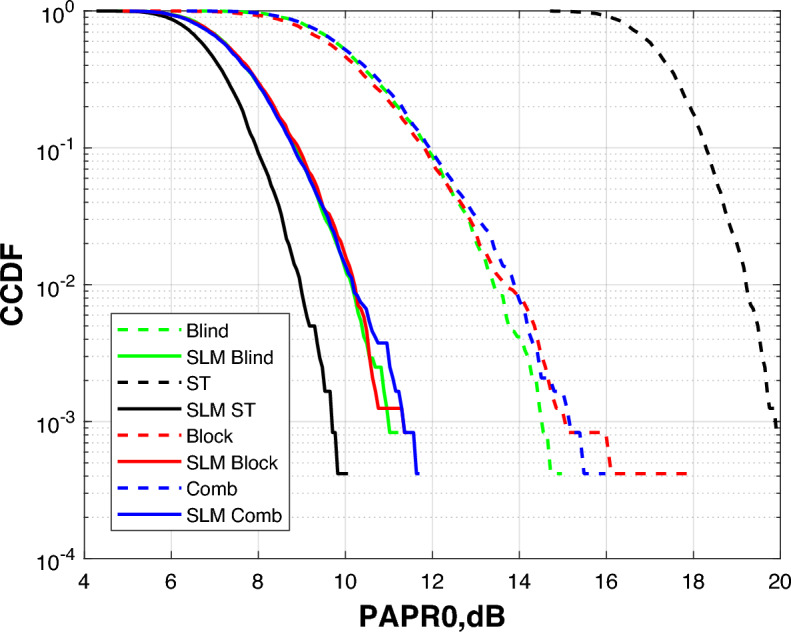
PAPR estimation of the DCO-OFDM-based VLC healthchare monitoring system using SLM technique of complex phase vector.

**Fig. 19:**
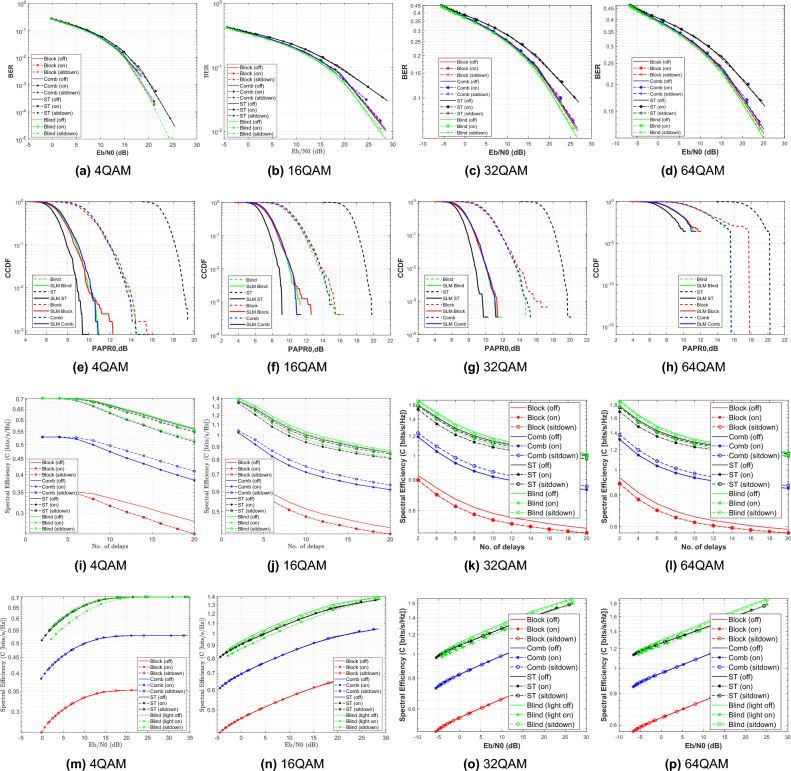
Comparison of BER, PAPR and spectral efficiency between different QAM orders.

Figure 18 demonstrates the system PAPR estimation with complex phase vectors in the SLM procedure. The results reveal a smaller further decrease in PAPR as compared to the real-valued phase vector scenario. This slight improvement implies that complex phase sequences offer more variety among potential OFDM signal blocks, raising the possibility of selecting a version with a minimum peak power. While the performance improvement is not significant, adding complicated phase vectors provides an additional degree of optimization, that can contribute to further decreasing nonlinear distortion in VLC transmitters, hence enhancing the energy efficiency and reliability of the biomedical healthcare monitoring system. We have selected QAM-4 and QAM-16 in this study to represent an optimal trade-off between BER and spectral efficiency. These values achieved adequate spectral efficiency and accepted BER. Accordingly, we tested also the proposed system using QAM-32 and QAM-64 as illustrated in Fig. 19.

Figure 19 demonstrates that as the order of QAM increases, the spectral efficiency increases, but the BER is degraded to unsatisfied values. On the other side, the PAPR values are unchanged by increasing the order of the QAM. In our study, BER and CCDF (of PAPR) are selected as the primary performance metrics because they directly reflect reliability and signal quality, which are critical for VLC-based healthcare monitoring systems. Nevertheless, additional system-level metrics such as latency, throughput, and power consumption are also relevant and can be evaluated. Latency can be defined as the end-to-end delay between data transmission and reception, including processing delay (modulation and encoding), transmission delay, and detection and decoding delay at the receiver. In simulation-based VLC systems, latency is typically estimated from parameters such as symbol duration, frame length, and signal processing complexity. However, these parameters were not fully modeled in our system, and therefore latency evaluation is beyond the scope of the present study. Throughput can be calculated as Throughput = Data Rate $\times$(1 - BER). Hence, improving BER directly enhances throughput, making BER an important indirect indicator of throughput performance. Finally, power consumption in VLC systems is closely related to PAPR, which explains the relevance of using the CCDF of PAPR as a performance metric. High PAPR increases LED nonlinearity and reduces power efficiency, whereas lower PAPR improves LED operating efficiency and reduces power consumption. Therefore, CCDF analysis provides an indirect yet effective measure of power efficiency.

The suggested OFDM-VLC healthcare monitoring system and a related study that also makes use of the OFDM modulation approach, but under optimal channel conditions, are compared in Table 3, in particular, a channel that is flat (without fading) and has no equalization. On the other hand, our solution uses four different equalization types and incorporates multipath fading to operate under challenging and realistic VLC channel circumstances.

**Table 3:** A brief Comparison with other OFDM related, systems.

Ref.	Mod	FFT Size	MCM (OFDM)	No. Sub.	Channel	Equalizer	PT	SNR(dB)	BER	PAPR (dB)	CCDF
^11^	16QAM	64	ADO	-	AWGN	-	u-law Mapping	25	$10^{-4}$	6.4	$10^{-4}$
^9^	16QAM 64QAM	-	ADO	-	AWGN	-	u-law Companding	26	$10^{-6}$	1.7	$10^{-3}$
^8^	QPSK	-	OFDM	128	AWGN	-	DCT-Gauss & VLM-Gauss	21	$10^{-6}$	9.2	$10^{-3}$
^5^	16QAM	1024	ASCO	16	AWGN	-	DST, DCT, DHT, & VLM	17.5	$10^{-4}$	13.4	$10^{-3}$
^6^	QPSK 16QAM	-	DCO	128	AWGN	-	SLM-MPC	-	-	6.8	$10^{-5}$
^13^	16QAM	16	ACO	-	LOS	-	DCT, DHT, VLM, WHT, & DST	15	$10^{-4}$	13.2	$10^{-3}$
^14^	4 QAM	-	DCO	64	AWGN	ML regressor (LPA)	-	30	$10^{-5}$	-	-
^15^	64 QAM	-	CAP-VLC	-	LOS-$1^{st}$ order NLOS	Hybrid CNN/DNN	-	20	2.1$\times 10^{-4}$	-	-
Proposed	QPSK 16QAM	64	DCO	48	AWGN &Fading	Block, Comb, ST & Blind	SLM	24 28 [0:30]	$$10^{-5}$$$$10^{-2}$$	10.1	$$10^{-4}$$

Despite the more rigorous simulation conditions in our study, we attained a PAPR of 9.6dB for 4QAM, and 10.2 dB for 16QAM, which is just slightly greater than the 9.5 dB PAPR obtained in the related work^8^ that considered a unity-gain, non-fading optical channel. This illustrates the resilience and efficiency of our system architecture, especially the application of SLM precoding algorithm and equalization techniques, which maintain performance in realistic and non-ideal installation settings of indoor biomedical healthcare monitoring environments.

## Computational complexity

The computational complexity is a key metric for evaluating the proposed system, quantified by the number of mathematical operations; including convolution, multiplication, and addition; executed at the transmitter and receiver. The number of subcarriers (*N*), the number of OFDM symbols ($n_{sym}$), and the number of SLM phase sequences *U* all have a significant impact on computational complexity. The multiplications count factor ($f_{mul}$) is computed as follows^7^:

35 $$\begin{aligned} f_{mul}= \frac{ n_{sym} ~ N ~ U }{2} ~ log_{2}( n_{sym} ~ N) \end{aligned}$$

Similarly, the additions count factor ($f_{add}$) is given by^7^:

36 $$\begin{aligned} f_{add}= n_{sym} ~ N ~ U~ log_{2}( n_{sym} ~ N) \end{aligned}$$

Moreover, the computational complexity reduction ratio (CCRR) for multiplication operations is defined as the ratio between the complexity of the proposed DCO-OFDM-based VLC system with SLM and that of the conventional system without SLM^42^:

37 $$\begin{aligned} CCRR (\%)=(1-\frac{f_{mul} ~(Proposed ~ System)}{f_{mul} ~ (Traditional ~ System)}) \times 100 \% \end{aligned}$$

The CCRR for addition operations are calculated in the same manner. The computation time ($t_{comp}$) is obtained from simulation results. When the computational complexities of the proposed and conventional systems are identical, CCRR equals zero. The CCRR increases as the complexity of the proposed system decreases relative to the conventional approach, reaching $100\%$ for negligible proposed complexity. Conversely, when the proposed system exhibits higher complexity, CCRR becomes negative, reaching $-100\%$ in the case of a twofold increase^42^. Table 4 present the calculated values using $n_{sym} =600$, $N =64$, and $U=3$. For the traditional system $U=1$ is assumed.

**Table 4:** Computational complexity, CCRR and computation time.

Complexity/System	$f_{mul}$	$f_{add}$	$t_{comp} ~ (sec)$
Tradional VLC System without SLM	292393	584786	22.23
Proposed DCO-OFDM-VLC with SLM System	877179	1754359	140
CCRR (%)	−200%	−200%	-

## Conclusion and future work

We concluded our work by using the requirement for reliable healthcare monitoring following the COVID-19 pandemic has highlighted the limitations of RF-based devices in medical situations. VLC, which provides inherent security and is resistant to radio frequency interference, is a great alternative. This research described a VLC system that uses DCO OFDM to offer reliable, high speed biomedical data transmission through indoor optical fading channel. Data was modulated using QAM with ordre 4 and 16. This study overcomes the high PAPR difficulty in DCO-OFDM-based VLC healthcare monitoring systems using Selected Mapping (SLM) precoding algorithm with 3 complex phase sequences, and 3 real sequences. Three common patient placement scenarios were used to assess four equalization techniques: block-type, comb-type, ST, and blind CE. A comprehensive comparative analysis of CE techniques was conducted under realistic patient positioning (LOS/NLOS) conditions. According to the results, SLM lowers PAPR by about 4 dB without causing BER deterioration. Blind CE outperforms other equalizers because of its ability of channel estimation without pilot requirement, delivering higher spectral efficiency. Further, simulating SLM using complex phase vectors gives an increase 1 dB of PAPR as compared to real-valued phase vectors, due to their greater phase variety. These results support the usefulness of combining SLM and blind CE for dependable, power-efficient biomedical healthcare monitoring VLC systems. Future work will incorporate actual medical data (e.g., ECG, SpO_2_), assess alternative precoding methods (DCT, DST, WHT), scale to multi-room hospital configurations, use deep learning-based CE, integrate NOMA for multi-user accessibility, and design energy and cost-efficient VLC equipment for wearable sensors.

For future work, it is recommended to practically execute the system and generate experimental results. Moreover, for advanced BER, suggest place the patient in an environment with lower effects or employ a hybrid enhanced equalizer.

## Data Availability

No clinical data is associated with this article. The data used and/or analyzed during the current study available from the corresponding author on reasonable request.

## References

[1] Fon, R. C., Ndjiongue, A. R., Ouahada, K. & Abu-Mahfouz, A. M. Fibre optic-VLC versus laser-VLC: A review study. *Photonic Netw. Commun.* **46**, 1–15 (2023).

[2] Tariq, F. et al. A speculative study on 6G. *IEEE Wirel. Commun.* **27**, 118–125 (2020).

[3] Vishwakarma, N. & Swaminathan, R. Performance analysis of hybrid FSO/RF communication over generalized fading models. *Opt. Commun.* **487**, 126796 (2021).

[4] Popoola, W., Gutema, T. Z. & Elamassie, M. Visible light communication VLC basics. In *Handbook of Radio and Optical Networks Convergence* 1–35 (Springer, 2024).

[5] Farid, S. M., Saleh, M. Z., Elbadawy, H. M. & Elramly, S. H. ASCO-OFDM based VLC system throughput improvement using PAPR precoding reduction techniques. *Opt. Quantum Electron.* **55**, 410 (2023).

[6] Aydin, V. & Hacioglu, G. Enhanced PAPR reduction in DCO-OFDM using multi-point constellations and DPSO optimization. *Neural Comput. Appl.* **36**, 5747–5756 (2024).

[7] Miriyala, G. & Mani, V. A new PAPR reduction technique in DCO-OFDM for visible light communication systems. *Opt. Commun.* **474**, 126064 (2020).

[8] Taha, B., Fayed, H. A., Aly, M. H. & Mahmoud, M. A reduced PAPR hybrid OFDM visible light communication system. *Opt. Quantum Electron.* **54**, 815 (2022).

[9] Hameed, S. M., Sabri, A. A. & Abdulsatar, S. M. A novel PAPR reduction method for ADO-OFDM VLC systems. *Opt. Quantum Electron.* **53**, 595 (2021).

[10] Shilpi, Shukla, M. & Kumar, A. PAPR reduction in OFDM for VLC system. In *Advances in VLSI, Communication, and Signal Processing: Select Proceedings of VCAS 2019*, 229–237 (Springer, 2020).

[11] Wang, T., Ren, Y., Li, C. & Hou, Y. A PAPR reduction scheme combining superimposed O-OFDM and -law mapping for VLC-OFDM systems. *Opt. Commun.* **460**, 125190 (2020).

[12] Zenhom, A. & Y., Hamad, E. K., Alghassab, M. & M. Elnabawy, M. Optical-ofdm VLC system: Peak-to-average power ratio enhancement and performance evaluation. *Sensors* **24**, 2965 (2024). 10.3390/s24102965 PMC11125151 38793820

[13] Zenhom, Y. A., Hamad, E. K. & Elnabawy, M. M. Throughput improvement in aco-ofdm-based vlc systems using noise cancellation and precoding techniques. *Opt. Quantum Electron* **56**, 1798 (2024).

[14] Salman, M. T., Siddle, D. R. & Udu, A. G. Machine learning approach to predict the DC bias for adaptive OFDM transmission in indoor Li-Fi applications. *IEEE Access*10.1109/ACCESS.2025.3527205 (2025).

[15] Li, Z. Deep learning-enhanced carrier less amplitude and phase modulation for high-speed visible light communication: mitigating led nonlinearity and bandwidth limitations. *Ain Shams Eng. J.* **16**, 103534 (2025).

[16] Elbakry, M. S., Mohammed, A. & Ismail, T. Throughput improvement and papr reduction for ofdm-based vlc systems using an integrated stc-imadjs technique. *Opt. Quantum Electron.* **54**, 418 (2022).

[17] Sobhy, A., ElSayed, S. & Zekry, A. Enhancing the performance of optical VLC system based on asymmetric symmetric subcarriers OFDM. *Int. J. Commun. Syst.* **33**, e4226 (2020).

[18] Ma, S. et al. Spectral and energy efficiency of ACO-OFDM in visible light communication systems. *IEEE Transactions on Wirel. Commun.* **21**, 2147–2161 (2021).

[19] Azim, A. W., Le Guennec, Y. & Maury, G. Performance analysis of precoded layered ACO-OFDM for visible light communication systems. *Opt. Commun.* **440**, 49–60 (2019).

[20] Panayirci, E., Bektaş, E. B. & Poor, H. V. Physical layer security with DCO-OFDM-based VLC under the effects of clipping noise and imperfect CSI. *IEEE Trans. Commun.*10.1109/TCOMM.2024.3367718 (2024).

[21] Kaushal, K. K., Yadav, V. & Jain, D. Enabling VLC data centers with DCO-OFDM technology. *J. Opt.*10.1007/s12596-024-01967-y (2024).

[22] Gurbilek, G., Koca, M. & Coleri, S. Blind channel estimation for DCO-OFDM based vehicular visible light communication. *Phys. Commun.* **56**, 101942 (2023).

[23] Karimullah, S., Sai Sumanth Goud, E. & Lava Kumar Reddy, K. Spectral efficiency for multi-bit and blind medium estimation of DCO-OFDM used vehicular visible light communication. In *ICDSMLA 2021: Proceedings of the 3rd International Conference on Data Science, Machine Learning and Applications*, 873–885 (Springer, 2023).

[24] Abed, G. A. A new approach to improve transmitting and receiving timing in orthogonal frequency division multiplexing OFDM systems. *Iraqi J. Comput. Sci. Math.* **4**, 83–96 (2023).

[25] Li, W. et al. Comparison and analysis of DC-biased OFDM channel estimation algorithms in VLC channel. In *2024 6th International Conference on Communications, Information System and Computer Engineering CISCE*, 117–121 (IEEE, 2024).

[26] Qian, X., Deng, H. & He, H. Pilot-based parametric channel estimation algorithm for DCO-OFDM-based visual light communications. *Opt. Commun.* **400**, 150–155 (2017).

[27] Kanwar, V., Sharma, D. & Thakur, H. Performance evaluation of block type and comb type channel estimation for OFDM system under various modulation techniques. *Performance Evaluation* **3**, (2013).

[28] Mahmoud, H. M., Mousa, A. S. & Saleem, R. Channel estimation based in comb-type pilots arrangement for OFDM system over time varying channel. *J. Netw.* **5**, 766 (2010).

[29] Sunkari, S. et al. Adaptive channel estimation using least mean square LMS for orthogonal frequency division multiplexing OFDM. In *2021 5th International Conference on Electronics, Communication and Aerospace Technology ICECA*, 579–585 (IEEE, 2021).

[30] Jiménez, J. C. E., Guzmán, B. G., García, M. J. F.-G. & Jiménez, V. P. G. Superimposed training-based channel estimation for visible light communications. In *2017 13th International Wireless Communications and Mobile Computing Conference IWCMC*, 240–245 (IEEE, 2017).

[31] Swaminathan, S. & Raajan, N. High-speed optical OFDM transmission by reducing the nonlinearity of LEDs in visible light communication systems. *Multim. Tools Appl.* **83**, 47353–47371 (2024).

[32] Jemimah, J., et al. An efficient PCCM masking scheme for PAPR reduction and encryption in OFDM-VLC system. *Telecommunication Systems* **87**, 167–197 (2024).

[33] Ullah, H., Sohail, M. & Bokhari, M. Dynamic range of LED in optical OFDM for PAPR performance analysis. *Opt. Quantum Electron.* **54**, 742 (2022).

[34] Sharan, N. & Ghorai, S. PAPR reduction and non-linearity alleviation using hybrid of precoding and companding in a visible light communication VLC system. *Opt. Quantum Electron.* **52**, 1–14 (2020).

[35] Sharifi, A. A. PAPR reduction of optical OFDM signals in visible light communications. *ICT Express* **5**, 202–205 (2019).

[36] Deepa, T., Suseela, V. & Mani, V. Performance analysis of novel precoding matrix techniques for optical OFDM-based visible light communication systems. *Opt. Laser Technol.* **154**, 108293 (2022).

[37] Gunturu, C. & Valluri, S. Joint PAPR reduction and channel estimation in low-complexity VLC systems. *Opt. Commun.* **570**, 130927 (2024).

[38] Hasan, M. M. VLM precoded SLM technique for PAPR reduction in OFDM systems. *Wirel. Pers. Commun.* **73**, 791–801 (2013).

[39] Huang, Y. et al. On improving the accuracy of visible light positioning system with SLM-based PAPR reduction schemes. In *2020 IEEE International Symposium on Broadband Multimedia Systems and Broadcasting BMSB*, 1–5 (IEEE, 2020).

[40] Uysal, M., Miramirkhani, F., Narmanlioglu, O., Baykas, T. & Panayirci, E. IEEE 802.15. 7r1 reference channel models for visible light communications. *IEEE Commun. Mag.* **55**, 212–217 (2017).

[41] Dimitrov, S. & Haas, H. *Principles of LED light communications: towards networked Li-Fi* (Cambridge University Press, 2015).

[42] Valluri, S. & Mani, V. A novel approach for reducing complexity in the slm-gfdm system. *Phys. Commun.* **34**, 188–195 (2019).

